# AFM nanoindentation reveals decrease of elastic modulus of lipid bilayers near freezing point of water

**DOI:** 10.1038/s41598-019-55519-7

**Published:** 2019-12-19

**Authors:** Calum Gabbutt, Wuyi Shen, Jacob Seifert, Sonia Contera

**Affiliations:** Clarendon Laboratory, Physics Department, University of Oxford, Parks Road, Oxford, OX1 3PU UK

**Keywords:** Membrane biophysics, Nanoscale biophysics, Atomic force microscopy, Biophysics, Biotechnology

## Abstract

Cell lipid membranes are the primary site of irreversible injury during freezing/thawing and cryopreservation of cells, but the underlying causes remain unknown. Here, we probe the effect of cooling from 20 °C to 0 °C on the structure and mechanical properties of 1,2-dipalmitoyl-sn-glycero-3-phosphocholine (DPPC) bilayers using atomic force microscopy (AFM) imaging and AFM-based nanoindentation in a liquid environment. The Young’s modulus of elasticity (*E)* at each temperature for DPPC was obtained at different ionic strengths. Both at 20 mM and 150 mM NaCl, *E* of DPPC bilayers increases exponentially –as expected–as the temperature is lowered between 20 °C and 5 °C, but at 0 °C *E* drops from the values measured at 5 °C. Our results support the hypothesis that mechanical weakening of the bilayer at 0 °C  is produced by  structural changes at the lipid-fluid interface.

## Introduction

Cryopreservation aims to preserve cells, tissues, organs and other biological constructs by cooling to very low temperatures so that they are capable of reanimation. Reliable, effective, quantitative cryopreservation science can transform medicine (e.g. fertility^1–3^, organ/tissue transplantation^4^, cell therapy^5^, regenerative medicine^6^), food security, animal husbandry^7–9^, genetic diversity, farming^10^, protection of endangered species^11^, resistance to zoonosis and even Mars colonisation programmes^12^.

The main challenge associated with cryopreservation is avoiding the damage to biological structures that occurs during both freezing and thawing at the phase transition of water around 0 °C. During the formation of ice, the removal of water presents severe challenges to the structural and functional integrity of biological systems. During cooling, energy within the system is removed and the self-association of water molecules leads to longer-lived intermolecular interactions that initiate the nucleation of ice microclusters, which then develop into larger ice crystals^13^. The ice crystal lattice cannot dissolve solutes, which are diverted into the residual liquid volume surrounding the ice interface. This decreases the freezing point of the residual water, which results in cells being exposed to gradually higher solute concentrations in a shrinking liquid water volume^14^. Residual mobile water persists down to very low temperatures^15^, contributing to the build-up of a osmotic stress that further affects cells. The physics of ice formation and melting is far from settled^16^. The liquid-to-solid nucleation of a supercooled aqueous solution is an example of a process of evolution of a metastable state to its final equilibrium state, whose physics is poorly understood. Such processes are some of the least understood phenomena in biology, physics, chemistry, and engineering^17^.

It is well established that solutions of molecular species can be supercooled, and hence it is possible to have water solutions of very high ionic concentrations that are well below 0 °C^18^. Furthermore ionic species and concentrations such as those present during cryopreservation of biological specimens have not been shown to induce ice nucleation^19^, which is one of the main problems that the field needs to deal with. On the other hand, it is well known that ice nucleation of biological samples occurs by heterogeneous nucleation mechanisms, i.e. biological interfaces, the walls of the containers can allow water molecules to take up configurations that allow the formation of ice^19^. Nucleation of ice is an uncontrolled variable in conventional cryopreservation leading to large variations between samples in the survival of cells after defrosting. It is now established that controlling the nucleation of ice is the most critical condition to improve protocols for cells for bio-banking. There are several techniques being used, such as seeding with small ice crystals, and adding nucleants such as pseudomonas syringae, crystalline cholesterol, or even silver iodide^19^.

Osmotic stress during freezing and thawing can lead to multiple types of cryoinjury^20–23^, such as the destabilization/mechanical rupture of cell plasma membrane^24–26^. Chemical cryoprotectants such as DMSO and ethylene glycol are often used to prevent cellular cryodamage^27^, but the high concentrations needed often lead to toxic effects^28^.

The cellular plasma membrane is responsible for the physical separation between the intra- and extracellular spaces. The maintenance of cell membrane integrity is an absolute minimum criterion for the selection of a successful cryopreservation process; and it is often used as the sole determinant of cell “viability”^29^. One of the main hypothesis to explain cryodamage of the membrane during freezing is dehydration of the lipid heads^30–33^. Recent work has suggested that changes at the interface of the lipid bilayer caused by small polar cyroprotectant molecules such as DMSO, glycerol or ethylene glycol^34,35^ could be responsible of changes in the physical properties of the resulting in resistance to cryodamage^36,37^.

Here, we use the unique capacity of AFM to image and test the mechanical properties of model system DPPC supported lipid bilayers in NaCl water solutions of different ionic concentrations as the temperature is lowered from 20 °C to 0 °C. DPPC bilayers have become one of the most used model system of phosphatidylcholine membranes in many biophysical studies, in particular DPPC is a good system to study the effect of lowering temperature on the mechanical properties of bilayers, since there exist previous validated data of the *E* of supported DPPC bilayers on mica surfaces, using AFM and other techniques^38,39^. The fact that supported bilayer properties differ from those of free-standing bilayers is often hypothesized as a limitation of supported bilayers experiments to explain biological situations. However, the plasma membranes of living cells are never “free-standing” they are usually locally supported and constrained by the cytoskeleton and the cytoplasm on one side, and the extracellular matrix on the other side. Local and global interactions between these biological supports and the molecules composing the membrane play an active role in the membrane function. Hence, experiments on supported lipid bilayers are arguably at least as biologically relevant as on free-standing membranes, and therefore the interaction of the bilayer with the substrate represents an opportunity to examine the influence of cell support on the local properties of the membrane. This is particularly important in AFM experiments since the nanoscale properties that we probe with this technique gives particular relevance to working to supported bilayers so that they are relevant for our understanding of natural systems^40^.

Here we use AFM-nanoindentation measurements to determine the elastic modulus (*E*) of the DPPC supported lipid bilayers during cooling from 20° to 0 °C. Our results are consistent with changes at the bilayer liquid interface resulting in mechanical weakening on the lipid bilayer at temperatures below 5 °C.

## Methods

### Sample preparation

1,2-dipalmitoyl-sn-glycero-3-phosphocholine (DPPC) was purchased from Avanti Polar Lipids. Stock solutions of DPPC/cholesterol dissolved in chloroform was stored at −6 °C. A 1 ml sample of this solution was placed into a glass vial and the chloroform was evaporated under a flow of N_2_ gas. A solution of NaCl of the desired ionic strength (20 mM and 150 mM) was added to the vial and the temperature of the sample was raised above the phase transition temperature of DPPC (41 °C); vesicles small unilamellar vesicles (SUV) were produced by sonication. Lipid bilayers were prepared on mica disks attached to a steel puck. Between 3–5 µl of the above solution was applied to freshly cleaved mica substrates, along with 100 µl of the appropriate NaCl solution. This was allowed to settle for 20 minutes, before the sample was rinsed with the NaCl solution. The samples were heated to 70 °C, allowing the formation of supported planar lipid bilayers on mica. The samples were then rinsed again and cooled down to room temperature.

### AFM imaging and nanoindentation

Imaging was done with an MFP-3D instrument (Oxford Instruments Asylum Research, Santa Barbara, CA) operated in amplitude-modulation mode (AM-AFM). In the experiments we used Olympus TR800 silicon nitride microcantilevers (nominal spring constant k = 0.57 N m−1, Olympus, Tokyo, Japan). The spring constant of each cantilever was determined using the thermal noise method^41^. Before imaging, the system was left to reach thermal equilibrium, this was monitored by checking that the deflection signal was stable. For each image, height, amplitude, and phase information were acquired simultaneously. For the calculation of the bilayer heights at each temperature, we used the distributions of pixel height of whole 256 × 256 images (which were previously plane fitted). The pixel distributions were Gaussian fitted to identify the position of the peaks and the standard deviation, which is plotted as error bars.

Bilayer nano-indentations for determination of *E* with AFM have been reported before by our group, and we follow a similar procedure here^38,42^. Force vs. distance curves were acquired on different areas of the lipid surface. Typically, more than 40 measurements were obtained for determining the mechanical properties presented at each temperature and ionic strength. Extension and retraction velocities were fixed to 100 nm s^−1^ for all measurements. For comparison, extension curves were also taken on mica before and after curve acquisition on lipid samples. The temperature of the sample during the experiments was controlled using a Peltier element and a temperature sensor incorporated into the AFM stage, which is accurate to 0.1 °C. Experiments started at 20 °C, and the temperature was lowered at a rate of 120 °C/minute. Before measurement at each temperature the system was allowed to reach thermal equilibrium.

### Calculation of elastic modulus E from indentation curves

Force curves taken in 5 °C intervals were fitted using the Bottom Effect Cone Correction (BECC) to the Sneddon model for a conical indenter^43^ which is applicable for thin samples for which the stiffness of the underlying substrate cannot be ignored. The tip is pyramidal at the micron scale, but for such small indentations (we only fit an area of <2 nm of the indentation curve) a conical indenter reproduces better the actual shape of the tip in this range. Using this model, the force *F* vs. indentation *δ* curve was fitted with the function:

$$F=\frac{8\,\tan (\alpha )E}{3\pi }{\delta }^{2}\{1+1.7795\frac{2\,\tan (\alpha )}{{\pi }^{2}}\frac{\delta }{h}+16{(1.7795\tan (\alpha )\frac{\delta }{h})}^{2}+O({\frac{\delta }{h}}^{3})\}$$

Where *α* is the half-angle of the cone (30 deg. was used from the manufacturer specifications) and *h* is the height of the sample which is calculated from the AFM images analysis. The Poisson’s ratio, *v*, is estimated to be 0.5, as for a perfectly incompressible material.

The fitting region was *δ* < 2 nm from the contact point with the sample. The contact with the sample was determined by a method that identifies the contact with the sample by calculating the point where the virtual work is minimized^44^. The curves were median filtered before fitting, which increased the signal-to-noise ratio, improving the contact point determination and the fitting.

## Results and Discussion

### Imaging and height analysis of DPPC planar lipid bilayers during freezing

**Figure 1:**
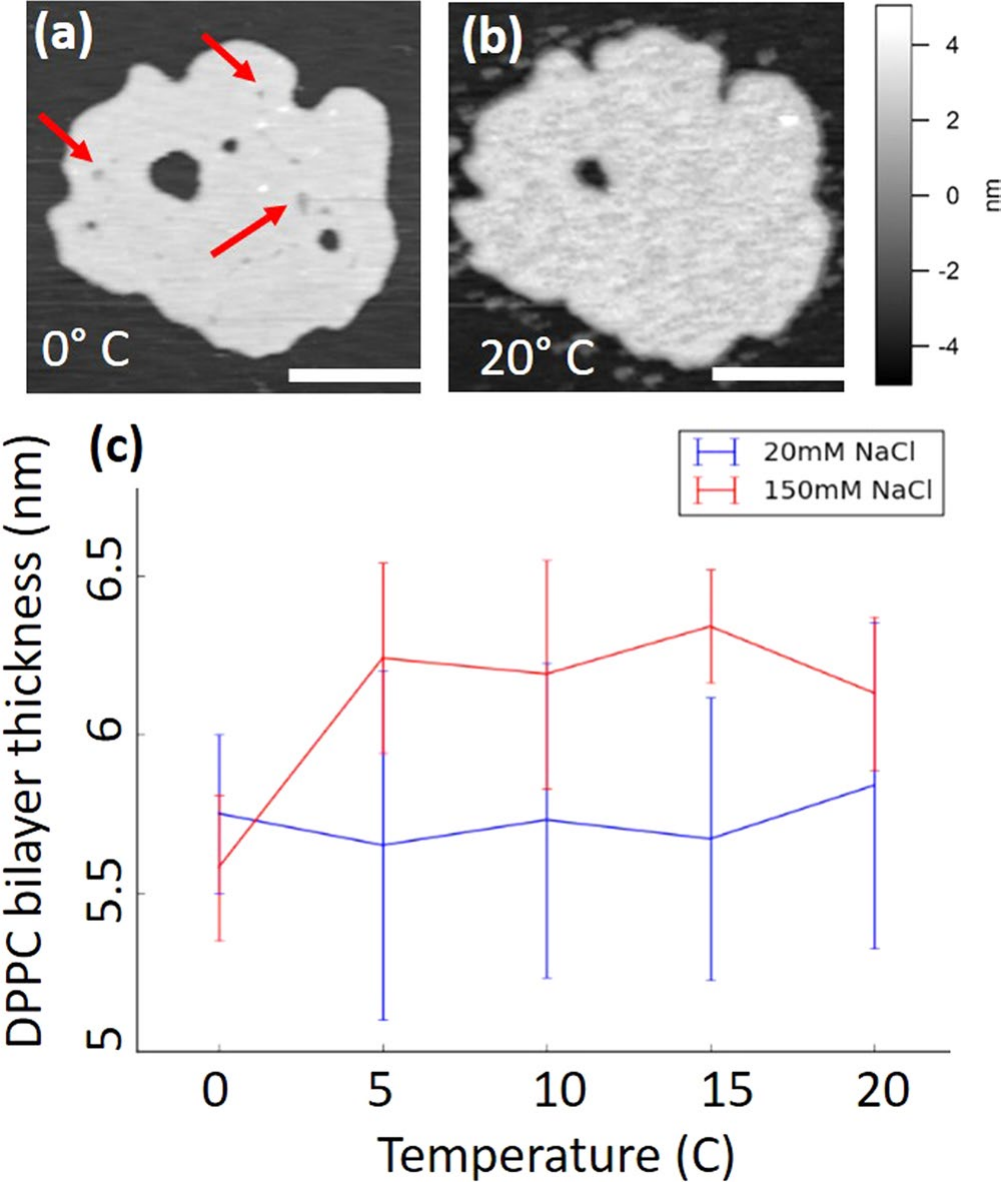
Effect of cooling on a DPPC supported bilayer structure. (**a**) Shows a typical AFM image of a DPPC lipid bilayer adsorbed on a mica substrate imaged in 20 mM NaCl at 0 °C, the red arrows indicate areas of the membrane where stable holes expanding half of the bilayer are visibles; (**b**) shows the same bilayer at 20 °C. (**c**) shows average lipid bilayer thickness data obtained by analyzing AFM images of samples at 20 mM and 150 mM NaCl solutions.

Supported DPPC lipid bilayers prepared as described in the Methods section were imaged using small amplitude AM-AFM in saline water solutions (20 and 150 mM NaCl) in the range of temperatures from 20° to 0 °C. Figure 1(a) shows a typical DPPC supported lipid bilayer on mica at 0 °C in a 20 mM NaCl solution. Figure 1(b) shows the same bilayer at 20 °C. At 0 °C the lipid bilayer presents small defects probably produced by lipid removed during scanning; smaller defects have a depth of between 1–2 nm (indicated with a red arrow), suggesting that the tip of the cantilever has cleaved only the top layer of the bilayer. The presence of these defects is probably due to a reduced lateral mobility of lipids at lower temperature, which prevents the disappearance of the defects. It is also likely that changes in the hydration shell of the lipids is also related to this weakening of the bilayer mechanical stability (see next section). These defects are not present at 20 °C.

In order to investigate the effect of ionic concentration on the height of the bilayers, we plot in Fig. 1(c) the average heights of lipid bilayers calculated from AFM images at 20 mM and 150 mM of NaCl obtained at 5 °C intervals from 20 °C to 0 °C. Error bars in this figure show 95% confidence intervals of standard deviation. At 20 mM NaCl there is no statistically significant correlation between the temperature and the thickness of the lipid bilayer; however, at 150 mM NaCl the bilayers compress by 0.7 ± 0.2 nm at 0 °C.

In the case of supported lipid bilayers on mica, the ions present at the interface between the membrane and the support can form a more ordered hydrogen bond network due to spatial confinement, which can affect properties such as e.g. the lipids transition temperature and molecular diffusion coefficients. The combination of electrostatics of the interacting hydration shells of both the dissolved ions and the head-groups are important in these interactions^40^. This effect would make it more unlikely for the lipids facing the mica surface to leave the bilayer during freezing, which would also explain the appearance of stable small holes expanding only the top leaflet of the bilayer at lower temperatures, as shown in Fig. 1 (indicated by red arrows).

### Lipid bilayer mechanical properties dependence on temperature: nanoindentation

In a typical AFM nanoindentation experiment the cantilever of the AFM is lowered towards the sample until the tip makes contact with it and the cantilever deflects upwards as the tip indents the sample. From the cantilever deflection vs. displacement curves it is possible to calculate the mechanical properties of the sample, such as *E*, using continuum mechanics models.

**Figure 2:**
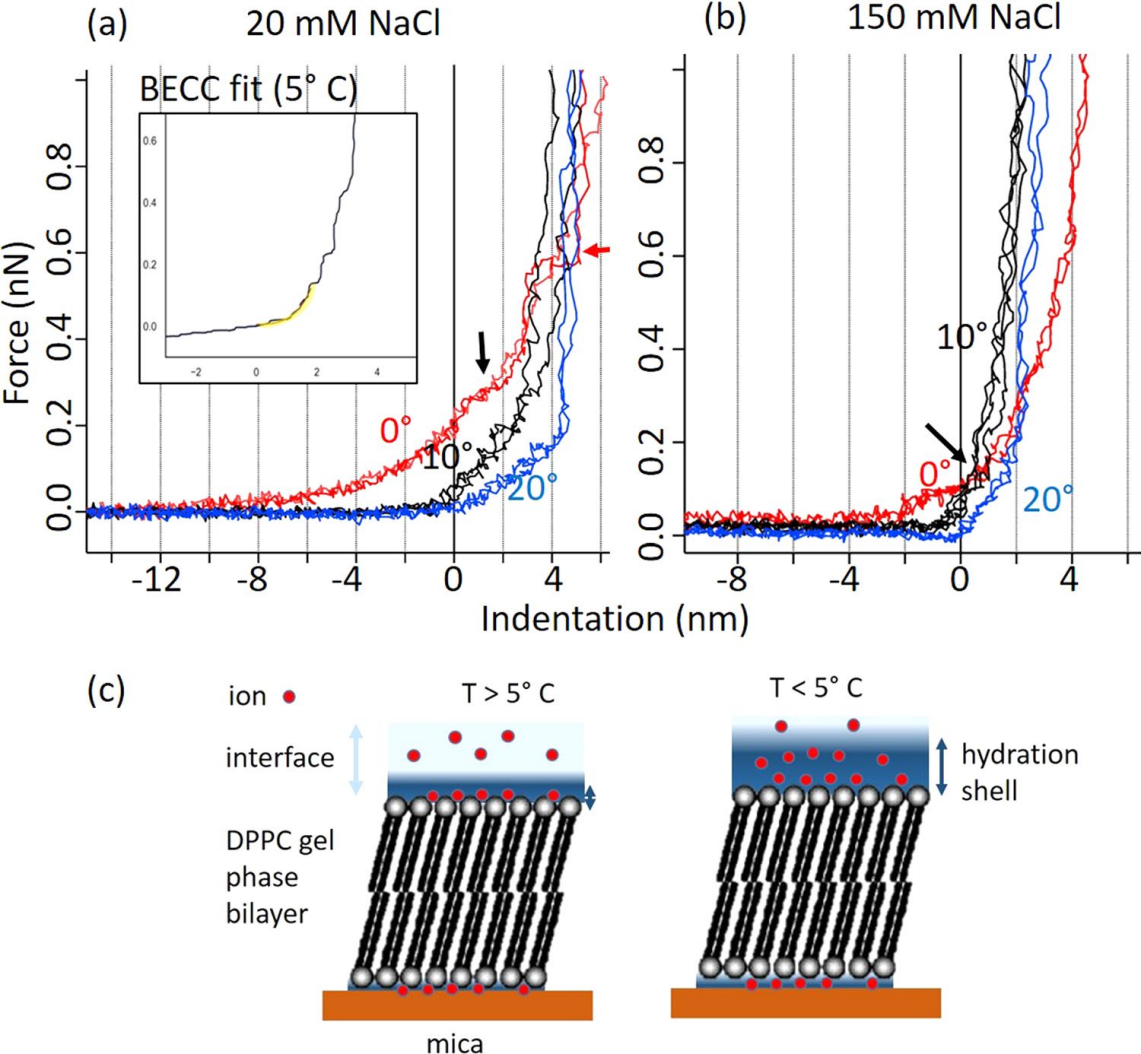
Force vs indentation curves for supported DPPC bilayers in 20 mM and 150 mM NaCl, at different temperatures. The contact point with the bilayer is marked by the zero in the x-axis. (**a**) shows force vs indentation curves of DPPC bilayers immersed in 20 mM NaCl solutions at 20° (blue), 10° (black) and 0°(red) C; (**b**) shows equivalent curves in 150 mM NaCl solution. The inset in (**a**) shows a typical indentation curve a 5 °C and the BECC theory fit to the curve after the contact point (yellow), the curve has been median filtered (see Methods). The red arrow in (**a**) shows a point where the tip is able to pierce the bilayer. The black arrows indicate initial indentation of the bilayer at 0 °C, showing larger deformation. (**c**) Shows a cartoon model of our hypothesis to explain the differences in the indentation curves before the contact point. At T < 5 °C the ions detach from the lipid head groups and create an extended hydration shell, while at T > 5 °C the hydration shell is shorter, and the ions create bridges between the lipid head-groups that increase the mechanical stability of the bilayer, making it stiffer.

In Fig. 2(a) we show typical force vs. indentation curves for DPPC lipid bilayers immersed in 20 mM NaCl and (b) in 150 mM NaCl water solutions, the figure compares curves obtained at 0°, 10° and 20 °C. The zero in the x-axis (indentation) marks the point of contact of the tip with the bilayer, the point of contact is found by making use of the fact that the maximum indentation cannot be higher than the total height of the bilayer, as previously reported for lipid membranes^42^.

At 0 °C, both at 20 mM and 150 mM NaCl concentrations, the indentation curves show a region of the liquid at the interface of the bilayer that is able to deflect the lever, which indicates that a hydration shell comprising hydrated screening ions, expands several nm at this temperature (see discussion below). The hydration shell is thicker at 20 mM than at 150 mM NaCl. Extended hydration shell regions at the interface of lipid membranes with the fluid that depend on salt concentration have been measured by AFM before^45^. In Fig. 2(c) we show a cartoon proposing how the shape of the indentation curves in the area before the contact with the bilayer would be related to the structure of the interface, ions would be in contact with the lipid heads at T > 5 °C, but they would detach at lower temperatures produced a more extended hydration shell.

After the cantilever tip makes contact with the bilayer, the slope of the curve increases as the tip indents the bilayer, and deforms it. It is clear that the slope is higher at 10° than at 20 °C in both cases indicating stiffening of the bilayer. In the case of 20 mM at 20 °C, the cantilever initially presses on the bilayer deforming it until it reaches an indentation of about 4 nm and then is not able to indent any longer and only manages to press the deformed bilayer on the mica substrate. At 10 °C the cantilever seems to be able to indent even less, and reaches the maximum indentation before 4 nm, this is consistent with a stiffer, a more cohesive bilayer as the temperature is lowered. However, at 0 °C, the cantilever is able to indent the bilayer over 5 nm. After the contact point with the bilayer at indentation zero, there is a change in slope (indicated with a black arrow), the slope here is lower that at 20 and 10 °C, indicating a softer leaflet. The red arrow in Fig. 2(a) indicates a point where the cantilever seems to pierce through the bilayer (or just the first leaflet), although rear, these events have been detected in a small percentage of curves in these conditions. All this information points towards a weakening of the bilayer, at 0 °C, which is easier to indent and deform that at higher temperatures. This behavior is confirmed with the appearance of holes in the bilayer observed in Fig. 1 at this temperature. It has been shown before that the absence of ions at a lipid membrane head-groups can result in the removal of lipids from one of the leaflets^42^.

The interpretation of Fig. 2(b) (data at 150 mM) is similar to the interpretation of Fig. 2(a), however in this case the hydration shell at the interface with the liquid at 0 °C is thinner. The thinning of the hydration shell at higher ionic concentrations, and at higher temperatures is consistent with a cloud of hydrated ions whose distance from the sample is determined by a competition between enthalpy and entropy and hence depends both on temperature and concentration of ions^46^. The maximum values of the indentation at 150 mM are smaller than at 20 mM, indicating more cohesive and stiffer layers at higher ionic concentrations.

In order to obtain the *E* of the bilayers, we fitted the 2 nm area of the indentation curve after the contact point (see Materials for details). An example of a fitted curve is given in the inset of Fig. 2(a). The lever that we have chosen for our experiments is not stiff enough to penetrate the bilayer (we do not observe any sudden jump indicating so, apart from a few curves at 0 °C at 20 mM NaCl, as indicated with the red arrow in Fig. 2(a)) and hence the cantilever continues to bend upwards after the initial 2 nm as the lipid bilayer is compressed against the underlying substrate. This effect is well known for the indentation of thin samples on hard substrates^43^. In order to obtain the mechanical properties of the bilayer we use the BECC correction of the Sneddon model (see Methods section) which takes into account the underlying hard substrate. We implement a recently developed algorithm to detect the contact point^44^; after the contact point is detected only the 2 initial nm of the indentation curve are fitted to the model, since beyond that, the BECC model would not be valid.

**Figure 3:**
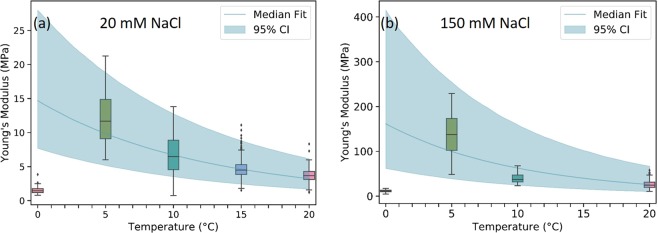
Boxplots of *E* vs. temperature for DPPC supported bilayers at (**a**) 20 mM NaCl and (**b**) 150 mM NaCl. The data are fitted to an exponential curve (see text) the shadow area marks the 95% confidence interval of the fit using Bayesian interference. In the boxplots, points further than 1.5 times the interquartile range away from the upper/lower quartile were labelled as outliers.

In Fig. 3(a) we show the calculated values of E and their dependence with temperature. The graphs show that *E* of DPPC bilayers increases as the temperature is lowered between 20 °C and 5 °C, but at 0 °C the Young’s modulus drops 80 ± 20% and 90 ± 50% at 20 mM and 150 mM NaCl respectively from the *E* values measured at 5 °C.

Since the error of the measurement is quite high, we will use the fact that between 20° and 5 °C the *E* of the bilayers increases exponentially at both salt concentrations (See Fig. S1 in the SI), to calculate the statistical significance of the results. The exponential dependence of mechanical properties with temperature has been previously demonstrated in phosphatidylcholine bilayers^47,48^. Following Niggemann^47^ and Pan^48^ from the fit to the exponential curve it is possible to obtain the bilayer flexibility activation energy *ε*_*k*_ (See SI). Form this fit we obtained value of *ε*_*k*_ = 8.4 ± 0.6 × 10^−20^J and *ε*_*k*_ = 4.8 ± 1.5 × 10^−20^J for DPPC in 20 mM and 150 mM of NaCl respectively, which is the first measurement to our knowledge reported for DPPC and coincides in magnitude with the flexibility activation energy of other phosphatidylcholine bilayers^48^. This is a further confirmation of the quantitative validity of our *E* measurements.

The exponential dependence of *E* vs. *T* allows us to use Bayesian statistical inference to show if the drop of E measured below 5 °C is statistically significant. In order to do this we fit the T > 0 data to the model log(E) ~ log(A) + m/T + N(0, sigma) with uninformative priors on the parameters A, m and sigma. Figure 3 shows an overlay the predicted E over the data with a 95% confidence interval (CI) for the fit; it is clear that the T = 0 data cannot be produced by this model, and hence we conclude that the reduction of *E* below 5 °C is demonstrated by our results.

In order to interpret this result we must note that our AFM experiments do not show freezing of the interface water at 0 °C. In fact, the AFM operation becomes impossible as ice forms, because the AFM operation stops immediately after ice formation, since the AFM tip probably acts as the nucleation site, which gets trapped in the ice and hence becomes incapable of imaging. This happens below 0 °C. It is likely that changes in the hydration of the polar lipid heads produced by the cooling are responsible for the decrease in *E*. We hypothesise that as water begins to pre-freeze the interactions between the lipid head-groups become weaker, resulting in a change in the free energy of the bilayers and consequently a decrease in *E*. The difference in the force curves at different salt concentration (Fig. 3) and the change in bilayer thickness at 0 °C for lipids in 150 mM NaCl (Fig. 1) indicate that electrostatic effects are also important in understanding the behavior at the lipid-water interface. Both the combination of reduced bilayer thickness and lower *E* could be caused by the depletion of immobile ions at the lipid bilayer surface, which are no longer able to bridge interactions between to the lipid heads. The large extended interfaces measured in the indentation curves at 0 °C for both salt concentrations seem to support this interpretation, the ions seem to detach from the immediate interface with the lipid heads (DPPC is zwitterionic) and form a less structured screening cloud (Fig. 2(c)). The lowering of temperature would change the relative contributions of entropic and enthalpic effects that determine the structure of the interface, which would result in a reduction of *E* at 0 °C.

Previous sub-nm resolution AFM studies have shown that water at the interface of a phosphatidylcholine lipid bilayer is ordered^49^ at room temperature, and also that ions order at the interface acting as bridges between the lipid heads^50^. Those AFM papers have established that the hydration shells of lipid bilayers are ordered and structured at room temperature in the immediate vicinity of the lipid heads. Our results seem to indicate that the order of the hydration shell of the bilayer might actually decrease at the interface as a result of cooling below 5 °C, resulting in a more fluid/glassy state that extends further into the bulk liquid that the tight ion ordered networks formed at the surface of the bilayer at higher temperatures. The reason for this is unclear. It is known that the thermal expansion coefficient of water becomes negative below 4 °C, i. e. water volume expands below 4 °C.^16^ This might have an effect in the observed results. Unlike simple liquids, fluctuations in liquid water increase upon cooling below a certain temperature instead of decreasing^16^. It is possible that changes in the structure of water below 5 °C increases the probability of ions leaving their positions as bridges of the lipid heads, creating a thicker, more disordered interface, and hence decreasing the elastic modulus of the bilayer as the interaction between the lipid heads weaken as a result.

## Summary and Conclusions

The mechanical effects of cooling planar lipid bilayer of DPPC from 20 °C to 0 °C were investigated at both physiological and low concentrations of NaCl (150 mM and 20 mM) using AFM imaging and nanoindentation. The thickness of DPPC bilayers immersed in 20 mM NaCl solutions does not significantly change with cooling temperatures, whereas at 150 mM the bilayers show a compression of 0.7 ± 0.2 nm at 0 °C.

Force-indentation measurements on lipid bilayers in solution were carried out at decreasing temperatures from 20 °C to 0 °C. As expected, at both 150 mM and 20 mM of NaCl, *E* was found to increase exponentially as the temperature was lowered from 20 °C to 5 °C. However, at 0 °C the lipid bilayers significantly soften. *E* drops 80 ± 20% for DPPC in 20 mM NaCl and 90 ± 50% in 150 mM NaCl from the values measured at 5 °C. Bayesian interference using the fact that the dependence of *E* vs *T* should be an exponential^47,48^, confirms that below 5 °C the bilayer does not follow the exponential behavior, and that the difference measured below 5 °C is statistically significant. The lowering of *E* below 5 °C coincides with the detection of a large hydration layer at the interface of the bilayer with the liquid, which expands beyond 5 nm at 20 mM NaCl, and is around 2 nm at 150 mM NaCl. The dependence on temperature and ionic concentration indicates that the thickness of this hydration shell is dependent on the balance of entropic and enthalpic contributions, and is likely related to the fact that the thermal expansion coefficient of water becomes negative below 4 °C, indicating that water volume expands below 4 °C, but further experiments are needed to confirm this interpretation. Our paper shows that AFM is a valuable tool to study the effect of lowering temperatures on the mechanical properties of lipid bilayers. This capacity is particularly useful in the context of cryopreservation as this technique can be used to investigate the effect of cryoprotectants such as glycerol or DMSO on the bilayer mechanical stability, as well as the effect of the membrane composition, and phase state.

## Supplementary material

Supplementary information
