# Supplementary material for: AFM nanoindentation reveals decrease of elastic modulus of lipid bilayers near freezing point of water
Source: Sci Rep. 2019 Dec 19;9:19473. doi: 10.1038/s41598-019-55519-7 (PMC6923397; doi:10.1038/s41598-019-55519-7)
Supplement: Supplementary file 1 — Supplementary information [file 41598_2019_55519_MOESM1_ESM.docx]

SUPPLEMENTARY INFORMATION

Title: **AFM nanoindentation reveals decrease of elastic modulus of lipid bilayers near freezing point of water**

Authors: Calum Gabbutt, Wuyi Shen, Jacob Seifert and Sonia Contera*

Content: **Exponential dependence of E vs T and calculation of flexibility activation energy for DPPC supported bilayers**

1. **Exponential dependence of E vs T and calculation of flexibility activation energy for DPPC supported bilayers**

It has been shown that the bending rigidity of a lipid bilayer, *k_c_*, has an exponential dependence with temperature (1, 2), and that follows the equation:

$k_{c}=Ae^{\frac{\varepsilon_{k}}{k_{B}T}}$ [s1]

where A is a constant, *k_B_* is the Boltzmann constant, and *ε_k_*  is the flexibility activation energy.

The values of *k_c_* at each temperature corresponding to our measurements of elastic modulus *E*, can be estimated using thin shell theory (3) using the equation:

$k_{c}=\frac{h^{3}E}{24\left( 1-\nu^{2} \right)}$ [s2]

where *h* is the height of the lipid bilayer, and $\nu$ is the Poisson’s ratio (value= 0.5).

Fig. S1 shows that our data follow equation [s1]. From the fit, using eq. [s1], we obtain

$\varepsilon_{k}$=8.4±0.6×10^-20^J and $\varepsilon_{k}$=4.8±1.5×10^-20^J for DPPC in 20mM and 150mM of NaCl respectively, which is the first measurement to our knowledge reported for DPPC and coincides in order of magnitude with the flexibility activation energy of other phosphatidylcholine bilayers (1, 2).


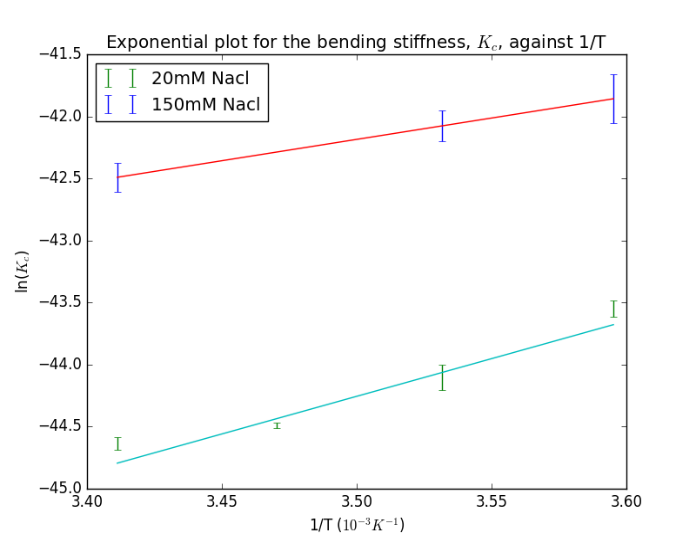


Fig S1. Arrhenius plot of the bending rigidity of DPPC bilayers vs *1/T* at 20 mM and 150 mM NaCl.

References :

1. Niggemann, G., Kummrow, M., Helfrich, W. (1995) The Bending Rigidity of Phosphatidylcholine Bilayers: Dependences on Experimental Method, Sample Cell Sealing and Temperature*. Journal de Physique II, EDP Sciences*, 5(3), 413-425.
2. Pan, J., Tristram-Nagle, S., Kucerka, N., Nagel, J. F. (2008). Temperature Dependence of Structure, Bending Rigidity, and Bilayer Interactions of Dioleoylphosphatidylcholine Bilayers. *Biophysical Journal*, 94, 117-124.
3. Picas, L., Rico, F., & Scheuring, S. (2012). Direct Measurement of the Mechanical Properties of Lipid Phases in Supported Bilayers. Biophysical Journal, 102(1), L01–L03.
